# Humanized Anti-hepatocyte Growth Factor Monoclonal Antibody (YYB-101) Inhibits Ovarian Cancer Progression

**DOI:** 10.3389/fonc.2019.00571

**Published:** 2019-07-09

**Authors:** Hyun Jung Kim, Sukmook Lee, Yong-Seok Oh, Ha Kyun Chang, Young Sang Kim, Sung Hee Hong, Jung Yong Kim, Young-Whan Park, Song-Jae Lee, Seong-Won Song, Jung Ju Kim, Kyun Heo

**Affiliations:** ^1^Research Institute, National Cancer Center, Goyang-si, South Korea; ^2^Department of Bioinspired Science, Ewha Womans University, Seoul, South Korea; ^3^Department of Applied Chemistry, Kookmin University, Seoul, South Korea; ^4^Department of Brain-Cognitive Science, Daegu-Gyeongbuk Institute of Science and Technology, Daegu, South Korea; ^5^Center for Uterine Cancer, National Cancer Center, Research Institute and Hospital, Goyang-si, South Korea; ^6^National OncoVenture, National Cancer Center, Goyang-si, South Korea; ^7^Clinical Research Team, Hanmi Pharm. Co., Ltd., Seoul, South Korea; ^8^Yooyoung Central Research Institute, Yooyoung Pharmaceutical Co., Ltd., Seoul, South Korea

**Keywords:** HGF, humanized monoclonal antibody, ovarian cancer, metastasis, YYB-101

## Abstract

Current chemotherapy regimens have certain limitations in improving the survival rates of patients with advanced ovarian cancer. Hepatocyte growth factor (HGF) is important in ovarian cancer cell migration and invasion. This study assessed the effects of YYB-101, a humanized monoclonal anti-HGF antibody, on the growth and metastasis of ovarian cancer cells. YYB-101 suppressed the phosphorylation of the HGF receptor c-MET and inhibited the migration and invasion of SKOV3 and A2780 ovarian cancer cells. Moreover, the combination of YYB-101 and paclitaxel synergistically inhibited tumor growth in an *in vivo* ovarian cancer mouse xenograft model and significantly increased the overall survival (OS) rate compared with either paclitaxel or YYB-101 alone. Taken together, these findings suggest that YYB-101 has therapeutic potential in ovarian cancer when combined with conventional chemotherapy agents.

## Introduction

Ovarian cancer has the highest mortality rate among gynecological diseases and is the fifth leading cause of cancer deaths among women in the US. The American Cancer Society estimated that in 2018, 22,240 women in the US were diagnosed with ovarian cancer and 14,070 died of this disease. The 5 year overall survival (OS) rate of patients with stage III or higher ovarian cancer is <29% (1). Because ovarian cancer progresses without symptoms, it is likely to have reached an advanced stage, with metastases to the peritoneal cavity, at initial diagnosis (2). The metastatic process of ovarian carcinoma is believed to involve the attachment of cancer cells shed by ovarian tumors to the surface of the peritoneum or to organs inside the peritoneum (3). Cancer cell seeding of the peritoneal cavity is associated with ascites, which ultimately progresses to high-grade carcinomas (4).

The standard treatment for ovarian cancer is cytoreductive surgery, followed by combination platinum and taxane-based chemotherapy (5). The most widely used combination is paclitaxel and carboplatin (6, 7). However, chemotherapy does not significantly improve the OS rate of patients diagnosed with advanced stage disease, mainly because of tumor resistance to chemotherapeutic agents (8). Efforts to improve clinical outcomes include co-treatment with targeted and chemotherapeutic agents. For example, the addition of bevacizumab, a monoclonal antibody against vascular endothelial growth factor (VEGF), or cetuximab, a monoclonal antibody against epidermal growth factor receptor (EGFR), was found to increase survival rates in patients with ovarian cancer in chemotherapy (9, 10). These findings suggest that targeted therapeutic agents may be effective and improve survival rates in patients with ovarian cancer.

Hepatocyte growth factor (HGF) is a scatter factor that promotes cell proliferation, migration, and invasion (11, 12). HGF binds to the HGF receptor c-MET, inducing several biological activities involved in cancer progression. The HGF/c-MET axis especially affects the migration of cancer cells from the primary site to other organs by promoting epithelial-mesenchymal transition (EMT), which initiates cancer cell metastasis (13, 14). HGF also stimulates the proliferation and inhibits the apoptosis of ovarian cancer cells, thereby enhancing cell survival (15, 16). Moreover, HGF concentrations were shown to be elevated in the ascitic fluid of ovarian cancer patients, suggesting that HGF enhances ovarian cancer cell migration and peritoneal dissemination (17, 18). In addition, c-MET inhibitors such as PF-2341066, foretinib and DCC-2701 have shown effective antitumor activities in ovarian cancer xenograft models (19–21).

YYB-101 is a humanized monoclonal antibody against HGF and a potential cancer treatment. Treatment of a mouse xenograft model of human colorectal cancer with a combination of YYB-101 and irinotecan, a chemotherapeutic agent used to treat colorectal cancer, effectively inhibited tumor progression (22). Treatment of a mouse xenograft model of human glioblastoma with YYB-101 and temozolomide (TMZ) resulted in a 2-fold higher survival rate than treatment with TMZ alone did (23). These findings suggested that combination therapy with YYB-101 and chemotherapeutic agents may inhibit tumor progression, including in ovarian cancer.

Therefore, in the present study, we assessed the effects of YYB-101 on ovarian cancer cells and a mouse xenograft model of ovarian cancer. The results of this study suggested that a combination of YYB-101 and conventional chemotherapeutic agents may be effective in treating patients with ovarian cancer by effectively inhibiting tumor metastasis and growth.

## Materials and Methods

### Cell Lines

The human ovarian cancer cell lines used in this study were the adenocarcinoma SKOV3, Caov-3, and OVCAR-3 (Korea Biotechnology Commercialization Center, KBCC; Incheon, Korea); A2780 (Sigma-Aldrich, St. Louis, MO, USA), and clear cell carcinoma JHOC-5 (Rikagaku Kenkyujyo, RIKEN, Tsukuba, Japan) cell lines. Cell culture media were obtained from Thermo Scientific Hyclone (Waltham, MA, USA). OVCAR-3, SKOV3, and A2780 cells were maintained in Roswell Park Memorial Institute (RPMI) medium supplemented with 10% heat-inactivated fetal bovine serum (FBS; Thermo Scientific Hyclone). Caov-3 cells were maintained in Dulbecco's modified Eagle's medium (DMEM) supplemented with 10% FBS. The JHOC-5 cells were maintained in DMEM/F12 medium supplemented with 10% FBS. All cells were grown at 37°C in a humidified incubator.

A2780 cells overexpressing firefly luciferase were generated as previously described (24). Briefly, A2780 cells were transfected with the firefly luciferase reporter plasmid pGL 4.51 (luc2/CMV/Neo; Promega, Madison, WI, USA) using Lipofectamine 2000 (Invitrogen, Waltham, MA, USA). The cells were cultured in medium containing 100 μg/ml G418 to select positive clones. The expression of luciferase was determined using a Dual-Luciferase reporter assay system (Promega) and luminescence was measured using a Victor luminometer (Perkin Elmer, Waltham, MA, USA).

### Cell Migration and Invasion Assays

YYB-101 was synthesized as described previously (25). Migration and invasion were assessed using 6.5 mm Transwell chambers with 8.0 μm pore filters (Costar, Cambridge, MA, USA). For migration assays, various concentrations of recombinant humanized HGF (R & D System, Minneapolis, MN, USA), YYB-101, paclitaxel (Corden Pharma Latina S.P.A, Sermoneta, Latina, Italy) or crizotinib (Sigma-Aldrich) were loaded into the lower compartment of each well, and SKOV3 (1 × 10^5^ cells per well) or A2780/luc (3 × 10^5^ cells per well) cells were placed on each Transwell insert. After incubation at 37°C for 8 or 48 h (SKOV3 or A2780 /luc cells, respectively), the inserts were removed and stained with a Diff-Quick staining kit (Sysmex, Kobe, Japan) and migrated cells were visualized using light microscopy.

For invasion assays, each Transwell plate was coated with 1 mg/mL Matrigel (BD Bio Sciences, San Jose, CA, USA), and each Transwell insert was filled with 2 × 10^5^ A2780/luc cells, 0.5 × 10^5^ SKOV3 cells, 1.5 × 10^5^ Caov-3 cells, or 1.0 × 10^5^ JHOC-5 cells. A2780/luc cells were incubated for 72 h while SKOV3, Caov-3, and JHOC-5 cells were incubated for 48 h. The inserts were stained with a Diff-Quick staining kit and invasive cells were visualized using light microscopy.

For drug combination experiments, synergistic effects were evaluated by the combination index (CI), which was calculated using CompuSyn software (ComboSyn, Inc., Paramus, NJ, USA). CI values were interpreted as follows: CI <1 indicated synergism, CI > 1 antagonism, and CI = 1 additive effect.

### Intraperitoneal Xenograft Mouse Model of Ovarian Cancer

All animal studies were approved by the Institutional Animal Care and Use Committee (IACUC, NCC-16-342) of the National Cancer Center, Republic of Korea. Luciferase-overexpressing A2780/luc cells (1 × 10^7^ in 200 μL phosphate-buffered saline [PBS]) were intraperitoneally injected into 7 week-old female BALB/c-nude mice (Orient bio, Korea). Three days later, the mice were intravenously injected with 40 mg/kg YYB-101 twice weekly and with 10 mg/kg paclitaxel once weekly. Ovarian cancer progression was monitored using bioluminescence imaging using the *in vivo* Imaging System (IVIS; Caliper Life Science, Waltham, MA, USA), and bioluminescence was quantified using Living Images software with identical standardized square regions of interest (ROI).

### Orthotopic Mouse Xenograft Model of Ovarian Cancer

Female BALB/C-nude mice were anesthetized with isoflurane and the right lateral sides of their abdomens were incised. The ovarian bursa was ejected, A2780/luc cells (1 × 10^5^ cells in 10 μL) were injected into the ovarian bursa, and the incision site was closed. One week later, the entire ovary of each mouse was excised and the mice were randomly grouped (*n* = 4–6 per group). Mice were injected intraperitoneally with 10 mg/kg paclitaxel once weekly and intravenously with 15 or 30 mg/kg YYB-101 twice weekly. Mice were monitored using the IVIS bioluminescence imaging device once weekly for 15 weeks.

### Statistical Analysis

All statistical analyses were performed using the GraphPad Prism software (GraphPad Software Inc., San Diego, CA, USA). The survival rate of xenografts was calculated using the Kaplan-Meier plots and compared using the log-rank test. Differences were analyzed with the Student's *t*-test or one-way analysis of variance (ANOVA) and a *p* < 0.05 was considered significant.

## Results

### HGF Enhances Metastasis of Ovarian Cancer Cells

The levels of HGF secreted by the A2780/luc, OVCAR-3, SKOV3, Caov-3, and JHOC-5 ovarian cancer cells into the culture media measured using enzyme-linked immunosorbent assay (ELISA) were 794,246.0, 90.5, 415.4, 1,140.4, and 368.1 pg/mL, respectively (Figure S1).

**Figure 1:**
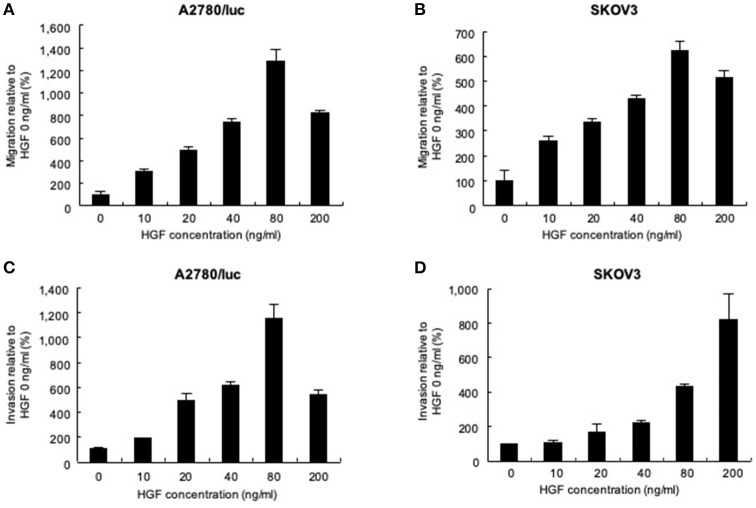
Effect of recombinant human hepatocyte growth factor (HGF) on the migration and invasion of ovarian cancer cells. Migration assays: **(A)** 3 × 10^5^ A2780/luc and **(B)** 1 × 10^5^ SKOV3 cells were seeded in upper chambers while the lower chambers contained 0, 10, 20, 40, 80, or 200 ng/mL HGF. After 48 or 8 h culture (A2780/luc or SKOV3 cells, respectively), migrated cells were observed using light microscopy. Invasion assay: **(C)** 2 × 10^5^ A2780/luc and **(D)** 0.5 × 10^5^ SKOV3 cells were seeded onto Matrigel-coated plates while lower chambers contained 0, 10, 20, 40, 80, or 200 ng/mL HGF. After 72 or 48 h culture (A2780/luc or SKOV3 cells, respectively), infiltrated cells were observed using light microscopy.

Migration and invasion assays were performed to determine whether HGF affects the movement of ovarian cancer cells. Using a Transwell system, we found that the migration (Figure 1A) and invasion (Figure 1C) of A2780/luc cells were maximal after treatment with 80 ng/mL HGF. The migration of SKOV3 cells was also maximal following treatment with 80 ng/mL HGF (Figure 1B), whereas invasiveness was HGF concentration-dependent (Figure 1D). In another experiment, we measured the HGF concentration of A2780/luc cells of the upper and lower chambers in the migration assay system without adding exogenous HGF to the lower chamber. As a result, we found that the concentration of HGF was gradually increased in the upper chamber after 8 h. However, the concentration of HGF was not detected in the lower chamber (data not shown). These data suggest that A2780/luc cells secrete HGF; however, the concentration and secretion period are not sufficient to affect the migration and invasion of the cells in this assay system. It is also possible that the initial concentration in the lower chamber is important to induce A2780/luc migration and invasion in this assay system.

### YYB-101 Inhibits Metastasis of Ovarian Cancer Cells *in vitro* and Suppresses Phosphorylation of C-MET

**Figure 2:**
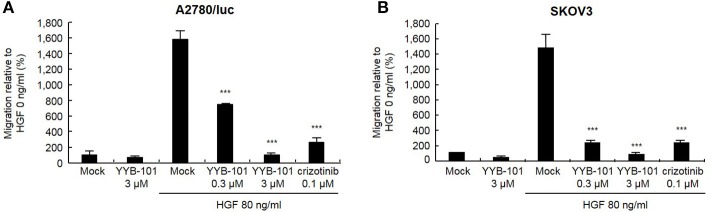
Effect of YYB-101 on migration of ovarian cancer cells. **(A)** A2780/luc (3 × 10^5^) and **(B)** SKOV3 (1 × 10^5^) cells were seeded in upper chambers. Lower chambers contained 3 μM YYB-101, 80 ng/mL Hepatocyte growth factor (HGF), 80 ng/mL HGF plus 0.3 μM or 3 μM YYB-101, or 80 ng/mL HGF plus 0.1 μM crizotinib. After culture for 48 or 8 h (A2780/luc or SKOV3 cells, respectively), migrated cells were observed using light microscopy. ^***^p < 0.001 vs. control, one-way ANOVA, followed by Tukey's multiple comparison test.

**Figure 3:**
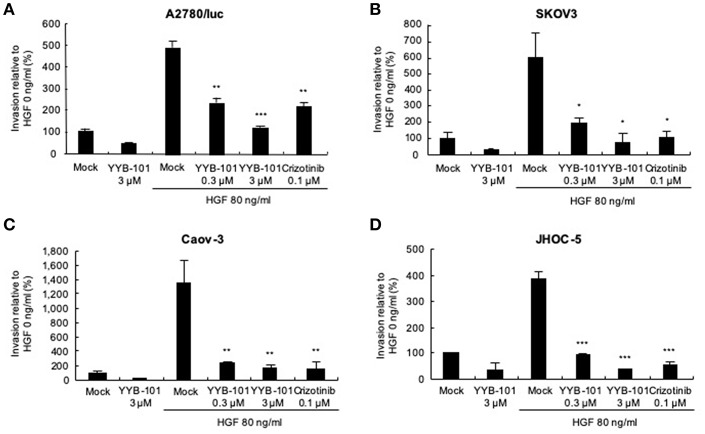
Effect of YYB-101 on invasion of ovarian cancer cells. **(A)** A2780/luc (2 × 10^5^), **(B)** SKOV3 (0.5 × 10^5^), **(C)** Caov-3 (1.5 × 10^5^), and **(D)** JHOC-5 (1.0 × 10^5^) cells were seeded onto Matrigel-coated upper chambers. Lower chambers contained 3 μM YYB-101, 80 ng/mL hepatocyte growth factor (HGF), 80 ng/mL HGF plus 0.3 μM or 3 μM YYB-101, or 80 ng/ml HGF plus 0.1 μM crizotinib. After culture for 72 h (A2780/luc cells) or 48 h (SKOV3, Caov-3, and JHOC-5 cells), infiltrated cells were observed using light microscopy; ^*^p < 0.05, ^**^p < 0.01, and ^***^p < 0.001 vs. control, one-way ANOVA, followed by Tukey's multiple comparison test.

**Figure 4:**
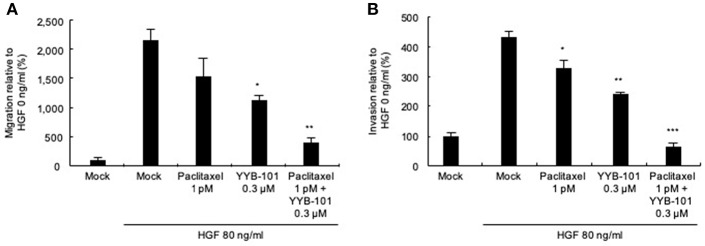
Efficacy of co-treatment with YYB-101 and paclitaxel on invasion of ovarian cancer cells. **(A)** A2780/luc (3 × 10^5^) cells were seeded in upper chamber. In the lower chamber, 1 pM paclitaxel, 0.3 μM YYB-101, or paclitaxel plus YYB-101 was added to the serum free culture media with 80 ng/mL hepatocyte growth factor (HGF). After 48 h culture, migrated cells were observed using light microscopy. **(B)** A2780/luc (2 × 10^5^) cells were seeded onto Matrigel-coated upper chambers. In the lower chamber, 1 pM paclitaxel, 0.3 μM YYB-101, or paclitaxel plus YYB-101 was added to serum free culture medium with 80 ng/mL HGF. After 72 h culture, migrated cells were observed using light microscopy. ^*^p < 0.05, ^**^p < 0.01, and ^***^p < 0.001 vs. control, one-way ANOVA, followed by Tukey's multiple comparison test.

We next tested the effect of YYB-101 on the migration and invasion of ovarian cancer cells. Cells were treated with 80 ng/mL HGF to induce migration, followed by treatment with 0.3 or 3 μM YYB-101 (Figure 2). The migration of A2780/luc and SKOV3 cells was reduced following treatment with 0.1 μM crizotinib, a small molecule inhibitor of lymphoma kinase (ALK), the c-ros oncogene (ROS1), and c-MET and was dose-dependently inhibited by YYB-101. The effect of YYB-101 on ovarian cancer cell invasiveness was tested using a Transwell system coated with Matrigel. Similar to its effects on migration, YYB-101 reduced the invasiveness of A2780/luc, SKOV3, Caov-3 and JHOC-5 ovarian cancer cells (Figure 3). Because paclitaxel is used as a first-line chemotherapeutics for patients with advanced ovarian cancer, we thought that co-treatment with YYB-101 and paclitaxel might have a synergistic effect on inhibition of invasion and migration of ovarian cancer cells. When A2780 cells were co-treated with paclitaxel and YYB-101, the migration, and invasion rates were effectively inhibited (Figure 4). These findings suggested that the HGF-induced migration and invasion of ovarian cancer cells was effectively inhibited by YYB-101.

The expression level of c-MET, the only known receptor for HGF, was assessed in SKOV3 cells using western blot analysis. In untreated and hIgG-treated SKOV3 cells, c-MET was phosphorylated on Tyr^1234/1235^; however, phosphorylated c-MET was not detected in YYB-101-treated SKOV3 cells (Figure S2A). In addition, treatment with YYB-101 reduced extracellular signal-regulated kinase (ERK) 1/2 phosphorylation, which occurs downstream of c-MET, compared with that in untreated or hIgG-treated cells. Furthermore, we found that c-MET phosphorylation was reduced in YYB-101-treated Caov-3 cells, as well as in SKOV3 cells (Figure S2B). Similarly, crizotinib treatment decrease c-MET activation at Tyr^1234/1235^ while PTX treatment did not.

Furthermore, some studies showed that HGF does not increase the viability of ovarian cancer cells (26, 27). We confirmed these results by WST-1 assay in SKOV3 and A2780 cells (Figure S3). Therefore, our results suggested that HGF-induced migration and invasion were not influenced by cell growth.

### YYB-101 Effectively Reduces the Progression of Ovarian Cancer *in vivo*

**Figure 5:**
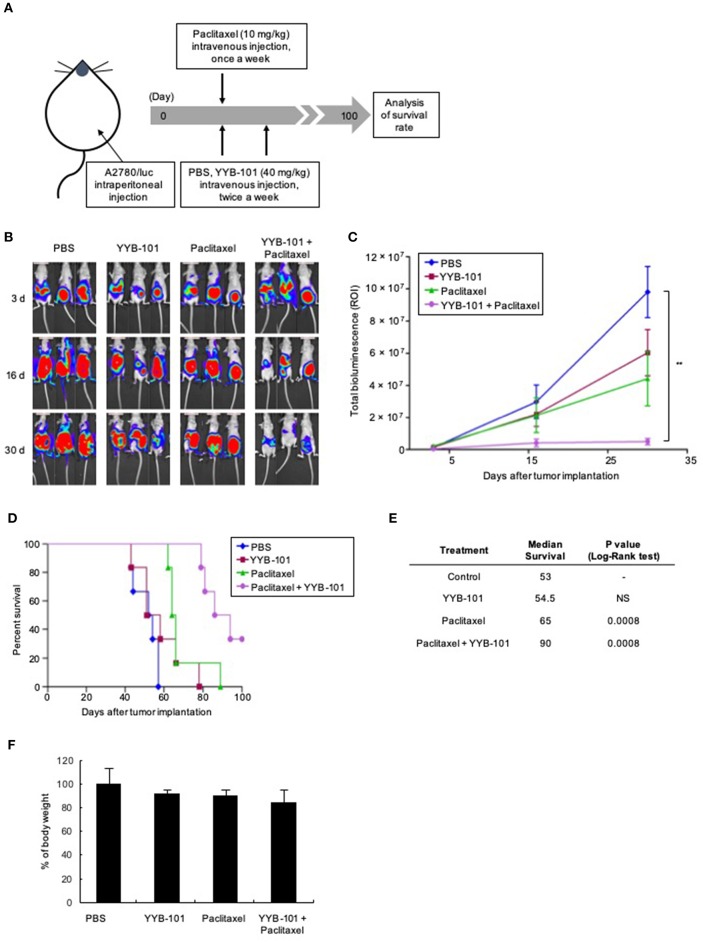
Efficacy of YYB-101 in a mouse ovarian cancer xenograft model. A2780/luc cells (1 × 10^7^) were intraperitoneally injected into mice, which were randomly divided into four groups (*n* = 6 per group). Control mice were injected intravenously with phosphate-buffered saline (PBS). Where indicated, mice were injected with 40 mg/kg YYB-101 twice weekly and with 10 mg/kg paclitaxel once weekly. **(A)** Schematic of experimental design. **(B)** Monitoring of tumor progression using *in vivo* Imaging System (IVIS) bioluminescence imaging once weekly. Mice were injected intraperitoneally with 75 mg/kg luciferin and bioluminescence images were obtained using an IVIS imaging device. Representative images 3, 16, and 30 days after tumor implantation are shown. **(C)** Quantitation of total bioluminescence using Living Image software. Error bars represent ± standard error of the mean (SEM). The *t*-test *p*-value was the comparison between co-treatment with control group (^**^p < 0.01). **(D)** Kaplan-Meier analysis of survival of various groups of mice for up to 100 days after cancer cell implantation. **(E)** Median survival was calculated using Kaplan-Meier statistic. Log-Rank, *p* < 0.001 for paclitaxel and combination treatment groups compared with control group. **(F)** Body weight change of mice for 30 days after tumor implantation. Values represent the mean ± standard deviation (SD). Average body weight of drug treatment groups was not significantly different compared with control group.

The effect of YYB-101 *in vivo* was examined using mouse xenograft models established by injecting A2780/luc cells intraperitoneally or implanting them orthotopically into the ovaries. In the intraperitoneal model, mice were treated with paclitaxel and YYB-101, starting 3 days after intraperitoneal injection, and bioluminescence imaging was performed weekly (Figure 5A). Following implantation of ovarian cancer cells, bioluminescence emission increased throughout the abdomens of control mice and those treated with YYB-101 or paclitaxel alone (Figure 5B). In contrast, bioluminescence emission was significantly reduced throughout the abdomens of mice co-treated with paclitaxel and YYB-101 (*p* < 0.01). Total bioluminescence (ROI) was also significantly lower in mice treated with paclitaxel and YYB-101 than in control mice (Figure 5C). Survival rates for up to 100 days after A2780/luc cell implantation were improved in mice treated with paclitaxel, YYB-101, or both compared with the control mice, with the OS rate being significantly higher in mice treated with paclitaxel and YYB-101 than in control mice (Figure 5D). The survival rate of the co-treatment group analyzed using the Log-Rank test was significantly (*p* < 0.001) different from that of the control group (Figure 5E). Change in body weight did not differ between mice treated with YYB-101 and control mice (Figure 5F). Statistical analyses of body weight changes showed no significant differences between each experimental group and the control group.

**Figure 6:**
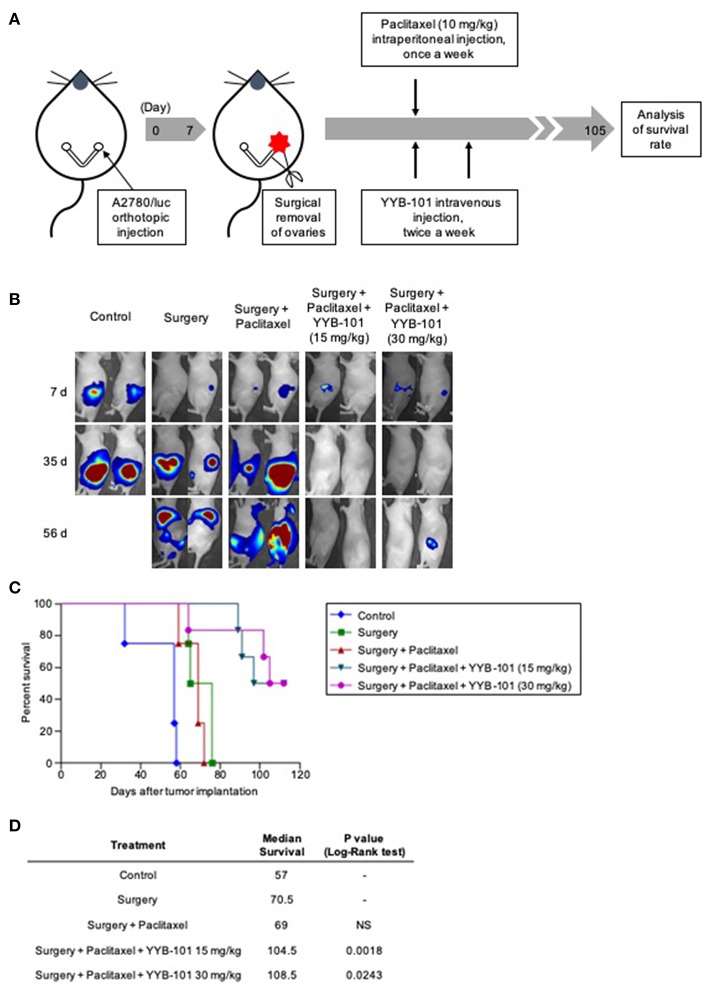
Efficacy of YYB-101 in mouse orthotopic xenograft of ovarian cancer. A2780/luc (1 × 10^5^) cells were injected into right ovaries of mice. One week after tumor implantation, ovaries were excised and mice (*n* = 4–6 per group) were treated with paclitaxel (10 mg/kg once weekly), alone or in combination with YYB-101 (15 or 30 mg/kg twice weekly). **(A)** Schematic of experimental design. **(B)** Mice were injected intraperitoneally with 75 mg/kg luciferin and bioluminescence images were obtained using the *in vivo* Imaging system (IVIS) imaging device. Representative images 7, 35, and 56 days after excision of ovaries are shown. **(C)** Kaplan-Meier analysis of mouse survival for up to 15 weeks after ovary excision. The *p*-value was calculated using Log-Rank test. **(D)** Median survival was calculated using Kaplan-Meier statics. Log-Rank, *p* < 0.05 for each group compared with the surgery only group.

Another *in vivo* mouse model, the orthotopic xenograft model, mimicked the treatment paradigm used to treat patients with ovarian cancer, consisting of cytoreductive surgery followed by paclitaxel treatment. The orthotopic xenograft model was designed to confirm the effect of paclitaxel and YYB-101 on cancer cells disseminated into the peritoneal cavity after cytoreductive surgery. We assessed the efficacy of YYB-101 in the orthotopic mouse xenograft model (Figure 6A) and co-treatment with YYB-101 and paclitaxel reduced the bioluminescence emission in the abdomen, whereas paclitaxel alone did not (Figure 6B). Mice treated with paclitaxel and YYB-101 had a significantly higher OS rate than mice treated with paclitaxel alone or control mice did (Figure 6C). We assessed whether co-treatment had a synergistic effect compared to single treatment, and used the Kaplan-Meier and Log-Rank test to analyze the statistically significant correlations with OS time. The result showed that the survival rate of the co-treatment group was prolonged more than that of the single treatment group (Figure 6D). These findings indicate that, when combined with a chemotherapeutic agent, YYB-101 can inhibit ovarian cancer progression *in vivo*.

## Discussion

The standard treatment regimen for patients diagnosed with ovarian cancer consists of surgery followed by chemotherapy. Conventional chemotherapy for ovarian cancer consists of combinations of carboplatin with taxane-based agents such as paclitaxel (6, 7, 28). Adjuvant chemotherapy was found to improve OS and recurrence rate in patients with early stage (I to IIA) ovarian cancer (29). However, 60–70% of patients are diagnosed with stage III or IV ovarian cancer or abdominal metastasis, and the recurrence rate in patients with advanced stage ovarian cancer is over 70% (7, 30). Therefore, it is important to provide new treatment options, such as new targeted therapy agents, for patients with advanced stage ovarian cancer.

Several co-treatments, including chemotherapeutic and targeted therapy agents, may overcome drug resistance and improve the efficacy of chemotherapy. Clinical trials have shown that bevacizumab, a monoclonal anti-VEGF antibody, administered alone or in combination with chemotherapy, was effective in patients with recurrent ovarian cancer (10). Because EGFR is overexpressed in up to 70% of ovarian cancers and is associated with poor prognosis, monoclonal anti-EGFR antibodies such as cetuximab have been added to paclitaxel and platinum-based agents in patients with recurrent ovarian cancer (9, 30). However, clinical trials of cetuximab in combination with paclitaxel and cisplatin showed only modest efficacy, including an 18 month progression free survival (PFS) rate of only 38.8% (9). The EGFR kinase inhibitor erlotinib and antagonistic antibody panitumumab have also shown limited success, improving PFS by 12.7 and 2.7 months, respectively (31, 32). Although several targeted agents, such as those against poly ADP ribose polymerase (PARP) and insulin-like growth factor receptor (IGFR), have been tested in clinical trials, they did not significantly improve PFS or OS (33, 34). New targeted agents are needed because current targeted agents have shown only limited success.

The results of the present study suggest that HGF has promise as a target molecule for the treatment of patients with ovarian cancer. HGF activates the c-MET signaling pathway, which stimulates the invasive and metastatic potential of various tumor cells (13, 14). The HGF/c-MET axis has recently become a therapeutic target for the treatment of various types of cancer. In several studies, c-MET inhibitors such as crizotinib and foretinib effectively inhibited the development and metastasis of ovarian cancer in animal models (19, 20, 35). HGF is expressed in normal ovarian epithelial cells and benign ovarian tumor cells, but to a higher degree in ovarian cancer cells (36, 37). Our results demonstrated that high levels of HGF were secreted by various ovarian cancer cell lines and HGF was involved in the increased migration and invasiveness of ovarian cancer cells.

YYB-101 was developed as a humanized neutralizing monoclonal antibody against HGF. YYB-101 was shown to inhibit HGF-induced scattering in MDCK-2 cells and block the phosphorylation of ERK, a downstream signaling molecule of c-MET that affects cell proliferation (23). YYB-101 was also found to be effective in various cancer models, including mouse xenograft models of colorectal cancer and glioblastoma, when coadministered with a chemotherapeutic agent (22, 23).

The present study verified the efficacy of YYB-101 in two mouse xenograft models of ovarian cancer, a widely used intraperitoneal model and a model where the ovaries were surgically removed after orthotopic xenografting. Although the efficacy of YYB-101 alone was similar to that of paclitaxel alone, their combination was highly effective, inhibiting the peritoneal progression of cancer cells and enhancing OS. These results suggested that co-treatment with YYB-101 and paclitaxel may significantly improve the survival rate by inhibiting the progression of ovarian cancer. paclitaxel induces the formation dysfunctional mitotic spindles, resulting in cell death through an apoptosis pathway. Despite of its activity, ~60% of patients with advanced stage ovarian cancer receiving standard treatment, consisting of cytoreductive surgery followed by chemotherapy with paclitaxel, experienced recurrence or drug resistance within 6 months, reducing their survival rate (38). However, we found that YYB-101 increased survival rate when combined with standard treatment, suggesting that adding YYB-101 to the standard treatment may improve outcomes.

In conclusion, the present study demonstrated that HGF was blocked by YYB-101, inhibiting the growth of ovarian cancer cells through the signaling pathway mediated by c-MET, the target receptor for HGF. However, although YYB-101 alone did not significantly inhibit metastasis of ovarian cancer, it showed synergistic effects with paclitaxel by inhibiting ovarian cancer progression *in vivo*. Further studies would be needed to elucidate the mechanism of the synergistic effect of combination therapy with YYB-101 and paclitaxel and to confirm the efficacy of YYB-101 in the xenograft model using various ovarian cancer cell types. Based on these results, co-treatment with YYB-101 and chemotherapeutic agents may overcome the limitations of chemotherapeutic agents alone, enhancing the treatment of advanced ovarian cancer. Currently, YYB-101 is undergoing Phase I (NCT02499224) clinical trials for solid cancers. The combination regimen of YYB-101 plus chemotherapy may benefit ovarian cancer patients who receive chemotherapy after cytoreductive surgery, which should be confirmed in Phase II clinical trials.

## Data Availability

All datasets generated for this study are included in the manuscript and/or the Supplementary Files.

## Author Contributions

Y-SO, SHH, JYK, S-JL, and KH discussed and designed the experiments. HJK, HKC, YSK, and JJK performed the experiments and analyzed the data. HJK, SL, Y-WP, S-WS, and KH discussed and wrote the paper.

### Conflict of Interest Statement

SHH is currently a full-time employee of Hanmi Pharm. Co., Ltd. S-JL, S-WS, and JJK are full-time employees of Yooyoung Pharmaceutical Co., Ltd. The remaining authors declare that the research was conducted in the absence of any commercial or financial relationships that could be construed as a potential conflict of interest.
