# Supplementary material for: Humanized Anti-hepatocyte Growth Factor Monoclonal Antibody (YYB-101) Inhibits Ovarian Cancer Progression
Source: Front Oncol. 2019 Jul 9;9:571. doi: 10.3389/fonc.2019.00571 (PMC6631954; doi:10.3389/fonc.2019.00571)
Supplement: Supplementary file 1 [file Data_Sheet_1.docx]

Supplementary Material

# Supplementary Data

## Figure S1. Detection of HGF secretion

To analyze the level of HGF secreted by ovarian cancer cells, 1 × 10^6^ cells were seeded into each well of a six-well plate and incubated overnight. The medium was replaced with medium containing 2% FBS and incubated for 48 h. The supernatant was collected and the concentration of HGF was measured using an HGF Quantikine ELISA kit (R&D Systems, Minneapolis, MN, USA).

## Figure S2. Immunoblot analysis

Antibodies used in immunoblot assays were purchased from Cell Signaling Technology, Inc. (Danvers, MA, USA). To assay the levels of c-MET and ERK1/2 expression and phosphorylation, 1 × 10^6^ SKOV or Caov-3 cells per well in RPMI medium or DMEM containing 2% FBS were seeded in six-well plates and cultured for 24 h. SKOV3 cells were replaced with serum-free medium, followed by the addition of 1 μM YYB-101 or hIgG and incubation for 24 h. Similarly, Caov-3 cells were replaced with serum-free medium, followed by the addition of 3 μM YYB-101, 1 pM PTX, 0.1 μM crizotinib, or 3 μM hIgG and incubated for 24 h. Cells were obtained and lysed, and whole cell lysates were subjected to western blot analysis using antibodies to c-MET, phospho-c-MET (Tyr1234/1235), ERK 1/2, phospho-ERK 1/2 (Thr 202/Tyr 204), and β-actin.

## Figure S3. Cell proliferation assay

SKOV3 (1.5 × 10^3^) or A2780 (3 × 10^3^) cells were seeded in a 96-well plate and incubated during overnight. After incubation, each cells were replaced with serum-free medium, followed by the addition of 0.3 μM YYB-101, 3 μM YYB-101, 1 pM PTX, 10 pM PTX, or 0.1 μM crizotinib. After 72 h of treatment administration, each cells were replaced with serum-free medium, and 10 μl WST-1 solution was added to each well and incubated for another 1 h. The absorbance of each well was obtained by VersaMax microplate reader (Molecular Devices, San Jose, CA, USA) at 450 nm.

# Supplementary Figures and Tables

## Supplementary Figures


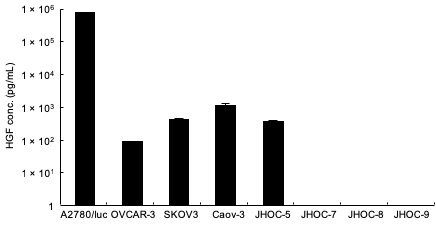


**Supplementary Figure 1.** Hepatocyte growth factor (HGF) secretion by ovarian cancer cells. A2780/luc, OVCAR-3, SKOV3, Caov-3, JHOC-5, JHOC-7, JHOC-8, and JHOC-9 cells (1 × 10^6^) were cultured in medium containing 2% FBS. After 48 h, HGF levels in the supernatants were measured using enzyme-linked immunosorbent assay (ELISA).


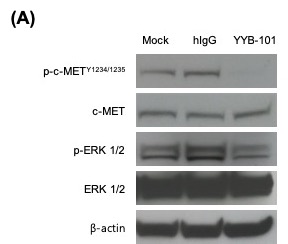

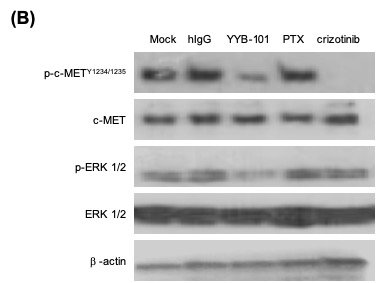


**Supplementary Figure 2.** Effect of YYB-101 on phosphorylation of c-MET and extracellular signal-regulated kinase (ERK) 1/2. (A) SKOV3 (1 × 10^6^) cells were treated with 1 μM YYB-101 or 1 μM hIgG (control). After 24 h, levels of c-MET and ERK 1/2 phosphorylation were determined using anti-phospho-c-MET (Y1234/1235) and anti-phospho-ERK 1/2 antibodies. (B) Caov-3 (1 × 10^6^) cells were treated with 3 μM YYB-101, 1 pM PTX, 0.1 μM crizotinib, or 3 μM hIgG (control). After 24 h, levels of phosphorylated c-MET and ERK1/2 were determined using anti-phospho-c-MET (Y1234/1235) and anti-phospho-ERK1/2 antibodies.


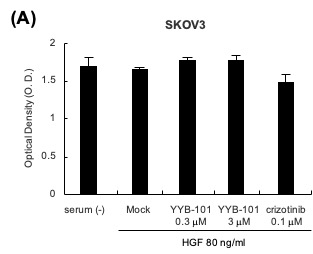

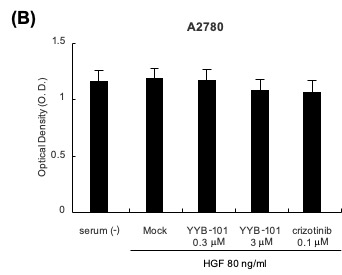


**Supplementary Figure 3.** Proliferation of HGF-treated SKOV3 and A2780 cells by WST-1 assay. (A) SKOV3 (1.5 × 10^3^) and (B) A2780 (3 × 10^3^) cells were seeded in 96-well plates and incubated overnight, after which the medium was replaced with serum-free medium containing 80 ng/ml HGF with 0.3 μM YYB-101, 3 μM YYB-101, or 0.1 μM crizotinib and incubated for another 72 h. The medium was replaced again with serum-free medium containing 10% WST-1 reagent and incubated for 1 h, followed by measurement of the supernatant (optical density at 450 nm) using a spectrophotometer.
